# Efficient (3*S*)-Acetoin and (2*S*,3*S*)-2,3-Butanediol Production from *meso*-2,3-Butanediol Using Whole-Cell Biocatalysis

**DOI:** 10.3390/molecules23030691

**Published:** 2018-03-19

**Authors:** Yuanzhi He, Feixue Chen, Meijing Sun, Huifang Gao, Zewang Guo, Hui Lin, Jiebo Chen, Wensong Jin, Yunlong Yang, Liaoyuan Zhang, Jun Yuan

**Affiliations:** 1 Key Laboratory of Biopesticide and Chemical Biology, Ministry of Education, College of Life Sciences, Gutian Edible Fungi Research Institute, Fujian Agriculture and Forestry University, Fuzhou 350002, China; Hyz19933@163.com (Y.H.); feixuechen@163.com (F.C.); 18950975897@163.com (M.S.); lovehuifang2012@163.com (H.G.); keepmovinggzw@163.com (Z.G.); lh4832@126.com (H.L.); jinws@fafu.edu.cn (W.J.); longyunyang@126.com (Y.Y.); 2 National Engineering Research Center for Sugarcane, Fujian Agriculture and Forestry University, Fuzhou 350002, China; jiebo-chen@fafu.edu.cn

**Keywords:** *meso*-2,3-butanediol, (3*S*)-acetoin, (2*S*,3*S*)-butanediol, *Escherichia coli*, coenzyme regeneration, whole-cell biocatalysis

## Abstract

(3*S*)-Acetoin and (2*S*,3*S*)-2,3-butanediol are important platform chemicals widely applied in the asymmetric synthesis of valuable chiral chemicals. However, their production by fermentative methods is difficult to perform. This study aimed to develop a whole-cell biocatalysis strategy for the production of (3*S*)-acetoin and (2*S*,3*S*)-2,3-butanediol from *meso*-2,3-butanediol. First, *E. coli* co-expressing (2*R*,3*R*)-2,3-butanediol dehydrogenase, NADH oxidase and *Vitreoscilla* hemoglobin was developed for (3*S*)-acetoin production from *meso*-2,3-butanediol. Maximum (3*S*)-acetoin concentration of 72.38 g/L with the stereoisomeric purity of 94.65% was achieved at 24 h under optimal conditions. Subsequently, we developed another biocatalyst co-expressing (2*S*,3*S*)-2,3-butanediol dehydrogenase and formate dehydrogenase for (2*S*,3*S*)-2,3-butanediol production from (3*S*)-acetoin. Synchronous catalysis together with two biocatalysts afforded 38.41 g/L of (2*S*,3*S*)-butanediol with stereoisomeric purity of 98.03% from 40 g/L *meso*-2,3-butanediol. These results exhibited the potential for (3*S*)-acetoin and (2*S*,3*S*)-butanediol production from *meso*-2,3-butanediol as a substrate via whole-cell biocatalysis.

## 1. Introduction

2,3-Butanediol (2,3-BD) is an important biobased bulk chemical with extensive applications in industry [1,2]. Its precursor, acetoin (AC), is one of the 30 platform chemicals which are given priority regarding their development and utilization by the United States Department of Energy [3,4]. 2,3-Butanediol has three stereoisomers ((2*S*,3*S*)-2,3-butanediol, (2*R*,3*R*)-2,3-butanediol, and *meso*-2,3-butanediol), while acetoin has two stereoisomers, including (3*R*)-acetoin and (3*S*)-acetoin [5,6,7,8]. These isomers of 2,3-butanediol and acetoin showed important potential for synthesis of pharmaceutical intermediates [6,7]. However, a mixture of 2,3-butanediol and acetoin isomers is generally produced by native producing strains, which results in difficulties for the production of acetoin and 2,3-butanediol with high chiral purity [6,9].

α-Acetolactate synthase (α-ALS), α-acetolactate decarboxylase (α-ALDC), and 2,3-butanediol dehydrogenase (BDH) in natural producing strains have been revealed in previous studies as three key enzymes involved in the biosynthesis of acetoin and 2,3-butanediol [10,11]. (3*R*)-Acetoin is mainly produced from pyruvate via α-acetolactate as one intermediate by α-ALS and α-ALDC. Meanwhile, the intermediate α-acetolactate is readily converted into diacetyl (DA) via non-enzymatic oxidative decarboxylation under oxygen supply conditions. Dependending on various BDHs with different stereospecificities, diacetyl can be catalytically converted into (3*S*)-acetoin and (3*R*)-acetoin. Ultimately, (3*S*)-acetoin and (3*R*)-acetoin can further be converted into three 2,3-butanediol isomer forms by BDHs, which show three types, including (2*S*,3*S*)-2,3-BDH, (2*R*,3*R*)-2,3-BDH and *meso*-2,3-BDH. During 2,3-butanediol fermentation process, *meso*-2,3-butanediol was synthesized from (3*R*)-acetoin by *meso*-2,3-BDH or (3*S*)-acetoin by (2*R*,3*R*)-2,3-BDH, whereas (2*S*,3*S*)-2,3-butanediol and (2*R*,3*R*)-2,3-butanediol were produced from (3*S*)-acetoin and (3*R*)-acetoin by (2*S*,3*S*)-2,3-BDH and (2*R*,3*R*)-2,3-BDH respectively [12,13]. These findings indicated that the existence of various 2,3-BDHs with different stereospecificities in native 2,3-butanediol producers leads to the formation of acetoin and 2,3-butanediol isomers during the fermentation process.

Based on the mechanism of acetoin and 2,3-butanediol isomers formation, metabolic engineering and synthetic biology have been applied in recent years to improve the productivity and purity. Zhang et al. developed a recombinant *E. coli* co-expressing α-ALS, α-ALDC from *Serratia marcescens* and NADH oxidase (NOX) from *Lactobacillus brevis* for (3*R*)-acetoin from glucose [14]. Maximum (3*R*)-acetoin concentration of 60.3 g/L with the purity of 97.3% could be achieved by fed-batch fermentative strategy. The highest (2*R*,3*R*)-2,3-butanediol concentration of 152.0 g/L with the purity of more than 97.5% was produced from a mixture of glucose and xylose using engineered *Enterobacter cloacae* strain through deleting *meso*-2,3-BDH and over-expressing (2*R*,3*R*)-2,3-BDH [15]. 98.0 g/L of *meso*-2,3-butanediol with the purity of 99.0% could also be obtained from glucose by *Bacillus licheniformis* deficient of glycerol dehydrogenase and acetoin degradable genes [16]. For (2*S*,3*S*)-2,3-butanediol production, Chu et al. introduced two genes encoding α-ALS and *meso*-2,3-BDH from *E. cloacae* into *E. coli*, which could produce low (2*S*,3*S*)-2,3-butanediol concentration (2.2 g/L) with a stereoisomeric purity of 95.0% from glucose using the fermentation method [17]. A major reason is that low concentration of diacetyl from α-acetolactate via non-enzymatic decarboxylation was produced during the fermentation process, resulting in low flux into (2*S*,3*S*)-2,3-butanediol via (3*S*)-acetoin by *meso*-2,3-BDH [18]. To obtain higher concentration and yield, biocatalytic technologies have been used to produce (3*S*)-acetoin and (2*S*,3*S*)-2,3-butanediol from diacetyl as a substrate. Li et al. employed whole-cell biocatalyst over-expressing *meso*-2,3-BDH from *E. cloacae* to convert diacetyl into (2*S*,3*S*)-2,3-butanediol via (3*S*)-acetoin as an intermediate, and glucose as a cheap source of reducing equivalents was used during the biocatalytic reaction process. High optical purity of (2*S*,3*S*)-2,3-butanediol (purity > 99%) with maximum concentration of 26.8 g/L was achieved using a fed-batch strategy [19]. The highest (2*S*,3*S*)-2,3-butanediol concentration of 31.7 g/L was obtained from diacetyl using 2,3-BDH with NADH-regeneration formate dehydrogenase (FDH) by in vitro biocatalysis [20]. Considering that (3*S*)-acetoin from diacetyl is readily converted into (2*S*,3*S*)-2,3-butanediol by BDHs, Gao et al. employed NADPH-dependent carbonyl reductase from *Gluconobacter oxydans* and glucose dehydrogenase from *Bacillus subtilis* with NADH regeneration to carry out the bioconversion from diacetyl to (3*S*)-acetoin [21]. Maximum (3*S*)-acetoin concentrations of 12.2 g/L were achieved from diacetyl by whole-cell biocatalysis. However, the toxicity of diacetyl to cells and enzymes was found to limit the biotransformation process and recyclability.

**Figure 1:**
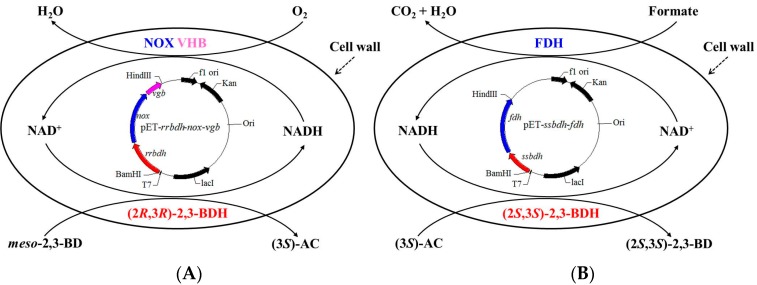
Schematic illustration of biocatalytic (3*S*)-AC (**A**) and (2*S*,3*S*)-2,3-BD (**B**) production from *meso*-2,3-BD using whole-cell biocatalysts of induced *E. coli* (pET-*rrbdh*-*nox*-*vgb*) cells and induced *E. coli* (pET-*ssbdh*-*fdh*) cells.

Previous studies indicated that *meso*-2,3-butanediol without diacetyl was also converted into (3*S*)-acetoin via one reversible reaction by (2*R*,3*R*)-2,3-BDH in the presence of NAD^+^ [22]. Xiao et al. showed that (3*S*)-acetoin could be efficiently produced from *meso*-2,3-butanediol using (2*R*,3*R*)-2,3-BDH from *Bacillus subtilis* coupled with NOX from *L. brevis* [23]. 36.7 g/L of (3*S*)-acetoin produced from *meso*-2,3-butanediol by whole-cell biocatalysis is still low. A possible reason is that the coenzyme regeneration system required oxygen as a substrate for NOX enzyme as described in our previous study [7]. In the current study, whole-cell engineered *E. coli* co-expressing (2*R*,3*R*)-2,3-BDH, NOX and *Vitreoscilla* hemoglobin (VHB) from *Paenibacillus polymyxa*, *L. brevis* and *Vitreoscilla* was developed for the production of (3*S*)-acetoin from *meso*-2,3-butanediol (Figure 1). Biotransformation conditions including pH, temperature, wet cell weight (WCW), substrate concentration, and metal ions were optimized in conical flasks. A high yield of (3*S*)-acetoin was obtained by batch bioconversion in 5-L bioreactor under the optimal conditions. Additionally, another biocatalyst co-expressing (2*S*,3*S*)-2,3-BDH and formate dehydrogenase (FDH) was developed and used to produce (2*S*,3*S*)-2,3-butanediol from (3*S*)-acetoin. Synchronous catalysis together with two biocatalysts showed that (2*S*,3*S*)-2,3-butanediol with high yield and purity could be achieved from *meso*-2,3-butanediol via (3*S*)-acetoin as intermediate.

## 2. Results and Discussion

### 2.1. Construction of the Whole-Cell Biocatalysts

**Figure 2:**
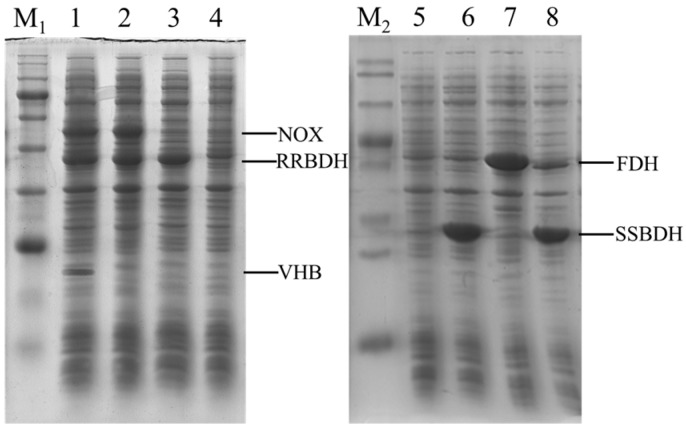
Expression analysis of recombinant *E. coli* strains by SDS-PAGE. Lane M_1_, marker proteins (180, 140, 100, 80, 60, 45, 35, 25, 15, 10 kDa); Lane 1, *E. coli* (pET-*rrbdh*-*nox*-*vgb*); Lane 2, *E. coli* (pET-*rrbdh*-*nox*); Lane 3, *E. coli* (pET-*rrbdh*); Lane 4–5, *E. coli* (pET28a); Lane M_2_, marker proteins (120, 100, 70, 50, 40, 30, 25, 14 kDa); Lane 6, *E. coli* (pET-*fdh*); Lane 7, *E. coli* (pET-*ssbdh*); and Lane 8, *E. coli* (pET-*ssbdh*-*fdh*).

First, three recombinant strains, including *E. coli* (pET-*rrbdh*), *E. coli* (pET-*rrbdh*-*nox*), and *E. coli* (pET-*rrbdh*-*nox*-*vgb*) were constructed for (3*S*)-acetoin production from *meso*-2,3-butanediol. SDS-PAGE analysis demonstrated that three clear bands of NADH oxidase, (2*R*,3*R*)-2,3-BDH, and VHB could be observed in three recombinant *E. coli* strains (Figure 2), implying that the *nox*, *rrbdh*, and *vgb* genes were successfully expressed or co-expressed in recombinant strains. Furthermore, the activities of (2*R*,3*R*)-2,3-BDH and NADH oxidase in the recombinant *E. coli* strains were determined, and *E. coli* (pET28a) was used as the control. The results were given in Table 1. As for (2*R*,3*R*)-2,3-BDH activity, the crude extracts from induced *E. coli* (pET-*rrbdh*), *E. coli* (pET-*rrbdh*-*nox*), and *E. coli* (pET-*rrbdh*-*nox*-*vgb*) cells exhibited the activities of 1.11, 0.97, and 0.95 U/mg for (2*R*,3*R*)-2,3-butanediol as substrate, and that of 0.57, 0.53, and 0.51 U/mg for *meso*-2,3-butanediol as substrate in the presence of NAD^+^ as coenzyme. No activity could be detected for (2*S*,3*S*)-2,3-butanediol as substrate by (2*R*,3*R*)-2,3-BDH. As described previously by Yu et al. [22], (2*R*,3*R*)-2,3-BDH from *P. polymyxa* could efficiently convert *meso*-2,3-butanediol and (2*R*,3*R*)-2,3-butanediol into (3*S*)-acetoin and (3*R*)-acetoin, respectively, whereas (2*S*,3*S*)-2,3-butanediol is not a substrate for (2*R*,3*R*)-2,3-BDH at all. In addition, the control strain *E. coli* pET28a also catalyzed the conversion of (2*R*,3*R*)-2,3-butanediol and *meso*-2,3-butanediol into acetoin though showing obviously low activity. Meanwhile, the NADH oxidase activities of 6.83 and 5.93 U/mg in *E. coli* (pET-*rrbdh*-*nox*) and *E. coli* (pET-*rrbdh*-*nox*-*vgb*) could be obtained when NADH was used as the substrate. Subsequently, the recombinant strain *E. coli* (pET-*ssbdh*-*fdh*) was developed for the conversion of (3*S*)-acetoin to (2*S*,3*S*)-2,3-butanediol. As shown in Figure 2, the *ssbdh* and *fdh* genes could be expressed or co-expressed in the recombinant *E. coli* strains. Moreover, enzyme activity assays were performed using the substrates of (3*R*/3*S*)-acetoin for (2*S*,3*S*)-2,3-BDH and formate for FDH. The crude enzyme from induced *E. coli* (pET-*ssbdh*-*fdh*) cells showed the activities of 38.90 U/mg for (3*R*/3*S*)-acetoin and 0.06 U/mg for formate (Table 2). These results indicated that whole-cell biocatalysts for the production of (3*S*)-acetoin and (2*S*,3*S*)-2,3-butanediol from *meso*-2,3-butanediol were successfully obtained.

### 2.2. (3S)-Acetoin Production from meso-2,3-Butanediol by Whole-Cell Catalysis

#### 2.2.1. Feasibility of (3S)-Acetoin Production from *meso*-2,3-Butanediol by Whole-Cell Catalysis

**Figure 3:**
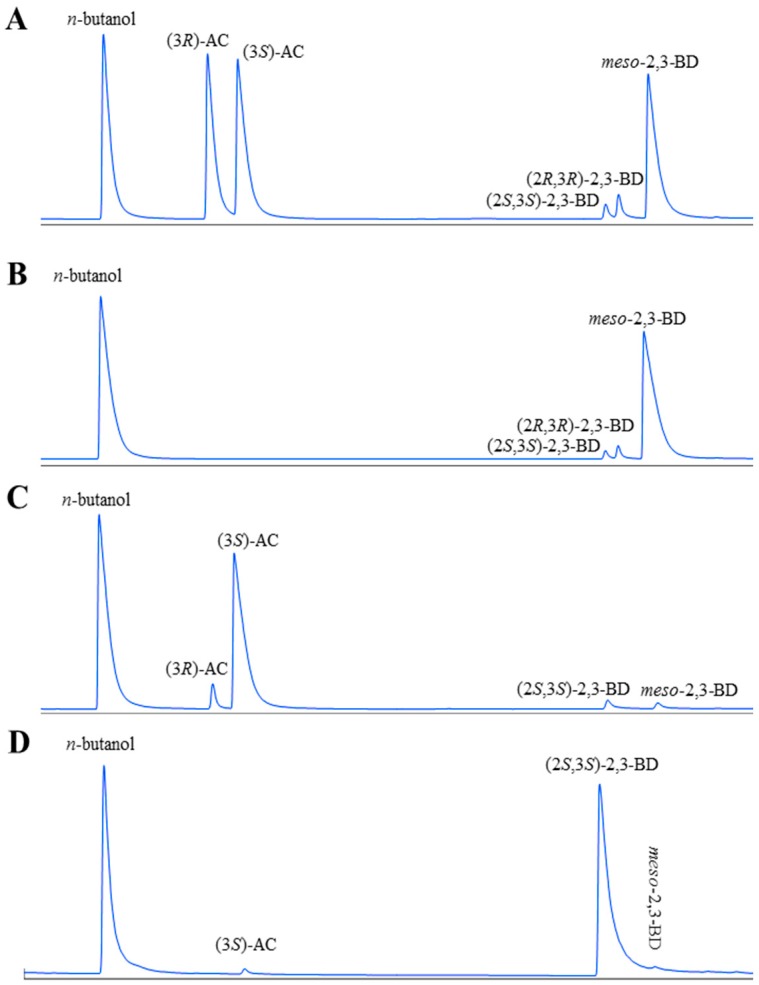
GC analysis of substrates and products in the bioconversion reaction by *E. coli* (pET-*rrbdh*-*nox*-*vgb*) and *E. coli* (pET-*ssbdh*-*fdh*) (*n*-butanol was used as the internal standard). (**A**) a mixture of standard chemicals of (3*R*/3*S*)-AC, (2*S*,3*S*)-2,3-BD, (2*R*,3*R*)-2,3-BD, and *meso*-2,3-BD; (**B**) the substrate of 2,3-BD (93.73% *meso*-2,3-BD, 2.23% (2*S*,3*S*)-2,3-BD, and 4.04% (2*R*,3*R*)-2,3-BD); (**C**) the product from the substrate of 2,3-BD by induced *E. coli* (pET-*rrbdh*-*nox*-*vgb*) cells; (**D**) the product from the substrate of (3*S*)-AC produced from *meso*-2,3-BD by induced *E. coli* (pET-*rrbdh*-*nox*-*vgb*) cells.

The whole-cell biocatalysis for (3*S*)-acetoin production from *meso*-2,3-butanediol was firstly conducted by using *E. coli* (pET-*rrbdh*), *E. coli* (pET-*rrbdh*-*nox*), and *E. coli* (pET-*rrbdh*-*nox*-*vgb*) in a 10 mL reaction mixture (pH 7.0) containing 20 g/L 2,3-butanediol and 40 g WCW/L at 30 °C for 12 h, and *E. coli* (pET28a) was used as the control. The configuration and yield of acetoin produced by whole-cell biocatalysis were analyzed and quantified using a GC system equipped with a chiral column. The results were shown in Figure 3 and Table 3. As shown in Table 3, 7.86 g/L of (3*S*)-acetoin from 20 g/L 2,3-butanediol as substrate was achieved by *E. coli* (pET-*rrbdh*). Meanwhile, a small amount of (3*R*)-acetoin (0.71 g/L) could be detected in the reaction system partially due to the existence of (2*R*,3*R*)-2,3-butanediol (4.04%) in the 2,3-butanediol substrate (Figure 3). As reported in previous studies, the (2*R*,3*R*)-2,3-BDH enzyme could convert (2*R*,3*R*)-2,3-butanediol into (3*R*)-acetoin, which resulted in a small amount of (3*R*)-acetoin production [13,22]. The (2*R*,3*R*)-2,3-BDH coupled with NOX enzyme in *E. coli* (pET-*rrbdh*-*nox*) resulted in the rapid increase of (3*S*)-acetoin yield (13.88 g/L), indicating that NAD^+^ regeneration could remarkably improve the whole-cell biocatalytic efficiency. Considering NOX enzyme required oxygen as substrate, the *vgb* gene encoding VHB enzyme introduced into *E. coli* (pET-*rrbdh*-*nox*) was employed to improve oxygen transfer during the whole-cell biocatalytic process [7,24]. As shown in Table 3, the expression of VHB led to an increase of (3*S*)-acetoin yield (16.79 g/L) by 20.97% compared with that by *E. coli* (pET-*rrbdh*-*nox*). The result was consistent with those of previous studies showing that VHB protein could be used to improve biocatalytic efficiency in biotransformation system with NOX-dependent NAD(P)^+^ regeneration [7,25]. 

Interestingly, a small amount of acetoin was also produced from 2,3-butanediol by the control strain *E. coli* (pET28a), which suggested that some non-specific dehydrogenases in *E. coli* might catalyze conversion of 2,3-butanediol into acetoin as observed in our previous study [13]. Based on the above results, the recombinant strain of *E. coli* (pET-*rrbdh*-*nox*-*vgb*) was chosen to perform the optimization of bioconversion conditions.

#### 2.2.2. Effects of pH, Temperature, and WCW on (3*S*)-Acetoin Production by *E. coli* (pET-*rrbdh-nox-vgb*)

**Figure 4:**
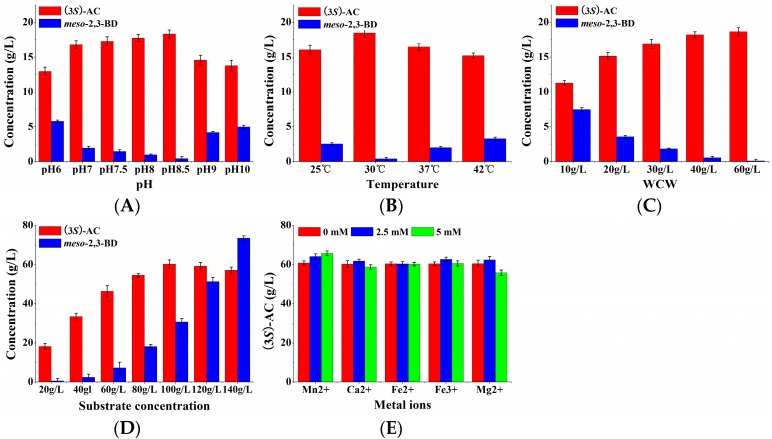
Effects of pH, temperature, WCW, substrate concentration and metal ions on (3*S*)-AC production from *meso*-2,3-BD by whole-cell biocatalysis of *E. coli* (pET-*rrbdh*-*nox*-*vgb*). (**A**) pH; (**B**) temperature; (**C**) WCW; (**D**) substrate concentration; (**E**) metal ions. The biocalytic reactions were performed in 100 mL flask containing 10 mL reaction solution for 12 h for optimization of pH, temperature and WCW in triplicate, while the optimizations of initial substrate concentration and metal ions were conducted in 100 mL flask containing 10 mL reaction solution for 24 h in triplicate.

To obtain high (3*S*)-acetoin concentrations, the effects of pH, temperature, and WCW on whole-cell biocatalytic efficiency were systematically investigated by using induced *E. coli* (pET-*rrbdh*-*nox*-*vgb*) cells. As shown in Figure 4A, the whole-cell biocatalytic reactions containing 20 g/L 2,3-butanediol and 40 g/L wet cells were conducted under different pH-values for 12 h at 30 °C. The results showed that the yield of (3*S*)-acetoin was gradually increased with the pH increase from 6.0 to 8.5. A maximum (3*S*)-acetoin concentration of 18.31 g/L could be obtained at pH 8.5. Thus, pH 8.5 as the optimum pH-value was used for the following experiments. The effect of temperature on (3*S*)-acetoin production from *meso*-2,3-butanediol by induced *E. coli* (pET-*rrbdh*-*nox*-*vgb*) cells was studied in the range of 25–42 °C. Figure 4B indicates that 18.45 g/L of (3*S*)-acetoin was achieved at 12 h when the whole-cell biocatalytic reaction was performed at 30 °C, which was used as the optimum temperature for further experiments. Among the parameters affecting whole-cell biocatalytic reaction, the concentration of the biocatalyst also plays an important role on the bioconversion efficiency [7,26], so WCWs from 10 to 60 g/L were used to evaluate the effect on the whole-cell biocatalysis process. As shown in Figure 4C, the (3*S*)-acetoin concentration was improved rapidly with the increase of WCW in the whole-cell biocatalytic reaction. The (3*S*)-acetoin concentration of 18.20 and 18.64 g/L could be produced from 20 g/L 2,3-butanediol by induced *E. coli* (pET-*rrbdh*-*nox*-*vgb*) cells at 12 h when the cell concentrations were 40 g and 60 g WCW/L. The WCW of 40 g/L was chosen as an optimum cell concentration after considering the cost.

#### 2.2.3. Effects of Substrate Concentration and Metal Ions on (3*S*)-Acetoin Production by *E. coli* (pET-*rrbdh-nox-vgb*)

Substrate concentration is another limiting factor in the whole-cell biocatalytic reaction. Low substrate concentration resulted in low product yield, whereas the bioconversion reaction might be inhibited by high substrate concentration. The effect of the initial 2,3-butanediol concentration on (3*S*)-acetoin production from *meso*-2,3-butanediol by *E. coli* (pET-*rrbdh*-*nox*-*vgb*) was tested in the range of 20–140 g/L for 24 h. The results indicated that the product (3*S*)-acetoin concentration was significantly increased by increasing the initial substrate concentration from 20 to 100 g/L (Figure 4D). When the initial 2,3-butanediol concentration continued to increase to 140 g/L, the (3*S*)-acetoin concentration appeared the decrease trend, implying that 100 g/L 2,3-butanediol as initial substrate concentration was suitable for (3*S*)-acetoin production. Ultimately, maximum (3*S*)-acetoin concentration of 60.29 g/L from 100 g/L 2,3-butanediol was obtained at 24 h (Figure 4D). Previous studies showed that the use of some metal ions such as Mn^2+^, Ca^2+^, Fe^2+^, Fe^3+^, and Mg^2+^ as chemical stimulators could accelerate the conversion from 2,3-butanediol to acetoin by improving the activity of (2*R*,3*R*)-2,3-BDH [22,27]. However, these metal ions showed stimulation or inhibition for NADH oxidase, another enzyme in the biocatalyst [7]. To evaluate their effect on (3*S*)-acetoin production, five metal ions including Mn^2+^, Ca^2+^, Fe^2+^, Fe^3+^, and Mg^2+^ at 0, 2.5, and 5.0 mM were chosen to conduct the whole-cell biocatalysis by using induced *E. coli* (pET-*rrbdh*-*nox*-*vgb*) cells for 24 h. The results showed that all the listed metal ions except Fe^2+^ could promote (3*S*)-acetoin production at different concentrations (Figure 4E). Especially Mn^2+^ exhibited obvious stimulation effect on (3*S*)-acetoin production in the bioconversion reaction. Maximum (3*S*)-acetoin concentration of 65.77 g/L was achieved by Mn^2+^ at 5 mM at 24 h.

#### 2.2.4. Batch Bioconversion of (3*S*)-Acetoin Production from *meso*-2,3-Butanediol in a 5-L Bioreactor

**Figure 5:**
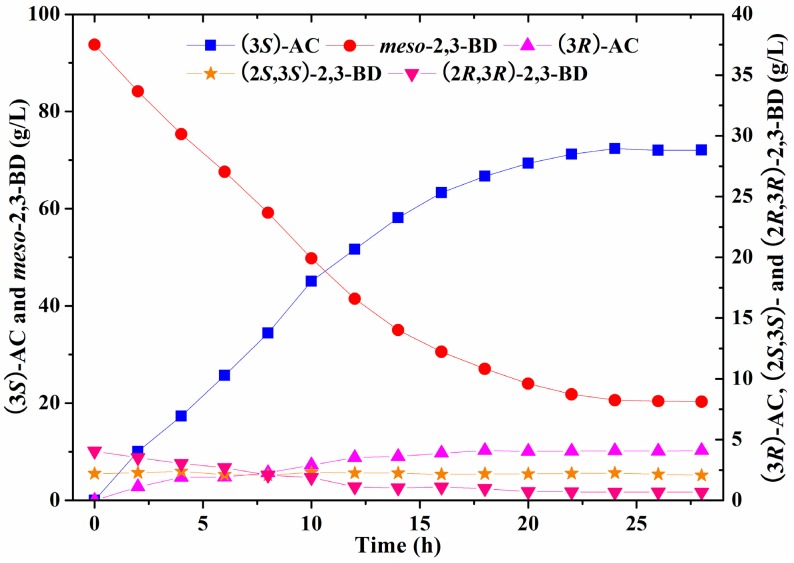
Time course of batch bioconversion of (3*S*)-AC production from *meso*-2,3-BD by *E. coli* (pET-*rrbdh*-*nox*-*vgb*) in 5-L bioreactor.

The batch bioconversion for (3*S*)-acetoin production from *meso-*2,3-butanediol by induced *E. coli* (pET-*rrbdh*-*nox*-*vgb*) cells was conducted in a 5-L bioreactor with the reaction volume of 2 L under the optimized conditions. The bioconversion experiment in the bioreactor was performed at 30 °C with an aeration rate of 4 vvm and agitation speed of 200 rpm to provide adequate oxygen. The result was shown in Figure 5. Maximum (3*S*)-acetoin concentration of 72.38 g/L with the productivity of 3.02 g/L/h and stereoisomeric purity of 94.65% was obtained by induced *E. coli* (pET-*rrbdh*-*nox*-*vgb*) cells at 24 h (Figure 5). However, the whole-cell biocatalyst could not completely convert *meso*-2,3-butanediol into (3*S*)-acetoin in the bioconversion reaction. A probable reason was that (2*R*,3*R*)-2,3-BDH was a reversible enzyme catalyzing the interconversion of *meso*-2,3-butanediol and (3*S*)-acetoin [13,22]. The residual *meso*-2,3-butanediol of about 20 g/L was retained in the whole-cell biocatalysis reaction partially due to the limitation of high (3*S*)-acetoin concentration. During the batch bioconversion process, (2*R*,3*R*)-2,3-butanediol from the substrate of 2,3-butanediol as another substrate could be converted into (3*R*)-acetoin by (2*R*,3*R*)-2,3-BDH, leading to the reduction of the (3*S*)-acetoin purity. Previous studies showed that high *meso*-2,3-butanediol concentration with a small amount of (2*S*,3*S*)-2,3-butanediol could be produced by *Serratia marcescens* H30, whereas no (2*R*,3*R*)-2,3-butanediol could be detected during the fermentation process [28,29]. Therefore, the substrate 2,3-butanediol without (2*R*,3*R*)-2,3-butanediol produced by *S. marcescens* H30 might contribute on high (3*S*)-acetoin purity in current reaction system. In addition, the (2*S*,3*S*)-2,3-butanediol concentration in the bioconversion system was kept at about 2.2 g/L since it was not the substrate for (2*R*,3*R*)-2,3-BDH in the biocatalyst.

### 2.3. (2S,3S)-2,3-Butanediol Production from meso-2,3-Butanediol by Synchronous Catalysis

#### 2.3.1. Feasibility of (2*S*,3*S*)-2,3-Butanediol Production from *meso*-2,3-Butanediol by Synchronous Catalysis

The (2*S*,3*S*)-2,3-BDH enzyme showed the ability of efficiently catalyzing (3*S*)-acetoin into (2*S*,3*S*)-2,3-butanediol in the presence of NADH in previous studies [13,30,31,32]. Herein, (2*S*,3*S*)-2,3-BDH from *Serratia* sp. T241 coupled with FDH from *C. boidinii* NCYC 1513 was employed to produce (2*S*,3*S*)-2,3-butanediol from (3*S*)-acetoin. As shown in Figure 1 and Figure 2, the biocatalyst of *E. coli* (pET-*ssbdh*-*fdh*) co-expressing (2*S*,3*S*)-2,3-BDH from *Serratia* sp. T241 and FDH from *C. boidinii* NCYC 1513 was developed for (2*S*,3*S*)-2,3-butanediol production from (3*S*)-acetoin produced from *meso*-2,3-butanediol by *E. coli* (pET-*rrbdh*-*nox*-*vgb*). Synchronous catalysis together with two biocatalysts was used to evaluate the feasibility of the conversion from *meso*-2,3-butanediol to (2*S*,3*S*)-2,3-butanediol via (3*S*)-acetoin as an intermediate (Figure 1). The initial reaction mixture contained phosphate buffer (pH 7.0), 20 g/L 2,3-butanediol, 20 g WCW/L of induced *E. coli* (pET-*rrbdh*-*nox*-*vgb*) cells, 20 g WCW/L of induced *E. coli* (pET-*ssbdh*-*fdh*) cells and 20 g/L ammonium formate as the driving force for NADH regeneration. The result showed that 13.96 g/L of (2*S*,3*S*)-2,3-butanediol could be achieved from 20 g/L *meso*-2,3-butanediol for 6 h at 30 °C by synchronous catalysis, implying that (2*S*,3*S*)-2,3-butanediol production from *meso*-2,3-butanediol was feasible by synchronous catalysis using two biocatalysts of induced *E. coli* (pET-*rrbdh*-*nox*-*vgb*) cells and induced *E. coli* (pET-*ssbdh*-*fdh*) cells.

#### 2.3.2. Optimization of Synchronous Catalysis Conditions

**Figure 6:**
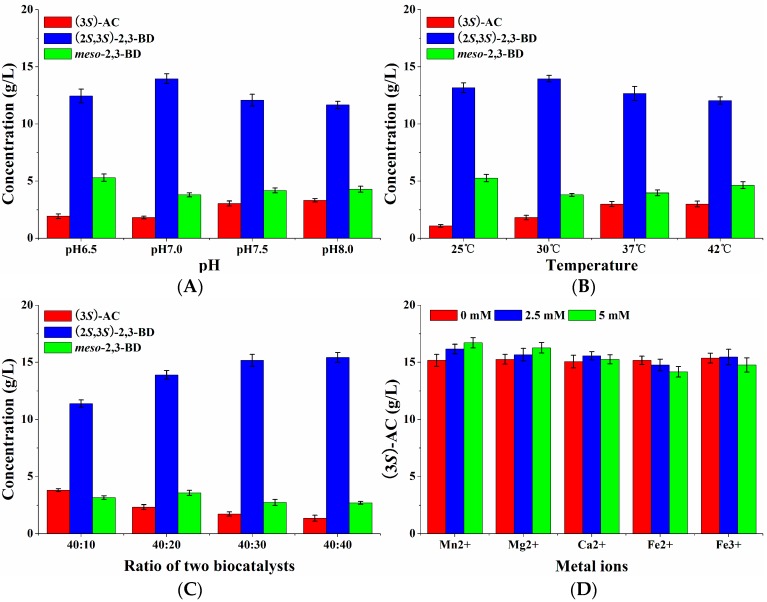
Effects of pH, temperature, ratio of induced *E. coli* (pET-*rrbdh*-*nox*-*vgb*) cells to induced *E. coli* (pET-*ssbdh*-*fdh*) cells, and metal ions on (2*S*,3*S*)-BD production from *meso*-2,3-BD by synchronous catalysis. (**A**) pH; (**B**) temperature; (**C**) ratio of *E. coli* (pET-*rrbdh*-*nox*-*vgb*) cells to induced *E. coli* (pET-*ssbdh*-*fdh*) cells; (**D**) different metal ions at the concentrations of 0, 2.5, and 5 mM. All the experiments were performed in triplicate in 100 mL flasks containing 10 mL reaction solution for 6 h.

Furthermore, four factors including pH, temperature, the WCW ratio of *E. coli* (pET-*rrbdh*-*nox*-*vgb*) cells to *E. coli* (pET-*ssbdh*-*fdh*) cells and metal ions were optimized using single factor experiments. The results were given in Figure 6. Figure 6A indicated that the pH value in the synchronous catalysis system had an important effect on (2*S*,3*S*)-2,3-butanediol production from *meso*-2,3-butanediol. The optimum pH was observed at 7.0, which was not consistent with that of (3*S*)-acetoin from *meso*-2,3-butanediol by *E. coli* (pET-*rrbdh*-*nox*-*vgb*). A probable explanation was that all the reported BDHs were reversible enzymes catalyzing the interconversion between acetoin and 2,3-butanediol. Alkaline environment favors the oxidation of 2,3-butanediol to acetoin by BDH enzymes, which readily reduced acetoin as a substrate to 2,3-butanediol in acid solution [5,13,22,27]. The effect of temperature on synchronous catalysis was investigated by using the optimum pH of 7.0. Two biocatalysts of induced *E. coli* (pET-*rrbdh*-*nox*-*vgb*) and *E. coli* (pET-*ssbdh*-*fdh*) cells exhibited high biocatalytic stability at 30 °C (Figure 6B). The ratio of *E. coli* (pET-*rrbdh*-*nox*-*vgb*) cells to *E. coli* (pET-*ssbdh*-*fdh*) cells was optimized at pH 7.0 and 30 °C as shown in Figure 6C. The results indicated that 15.17 and 15.43 g/L of (2*S*,3*S*)-2,3-butanediol production from 20 g/L *meso*-2,3-butanediol could be achieved at 6 h when the ratios of *E. coli* (pET-*rrbdh*-*nox*-*vgb*) to *E. coli* (pET-*ssbdh*-*fdh*) were 40:30 and 40:40 respectively. To consider the cost, the ratio (40:30) of two biocatalysts was chosen for following experiments. The effect of metal ions with different concentrations on synchronous catalysis was shown in Figure 6D. The results showed that Mn^2+^ among all the listed meal ions played an obvious stimulation effect on (2*S*,3*S*)-2,3-butanediol production from *meso*-2,3-butanediol in the synchronous catalysis reaction. Maximum (2*S*,3*S*)-2,3-butanediol concentration of 16.71 g/L was obtained in the presence of 5.0 mM Mn^2+^ for 6 h.

#### 2.3.3. Batch Bioconversion of (2*S*,3*S*)-2,3-Butanediol Production from *meso*-2,3-Butanediol by Synchronous Catalysis in 5-L Bioreactor

**Figure 7:**
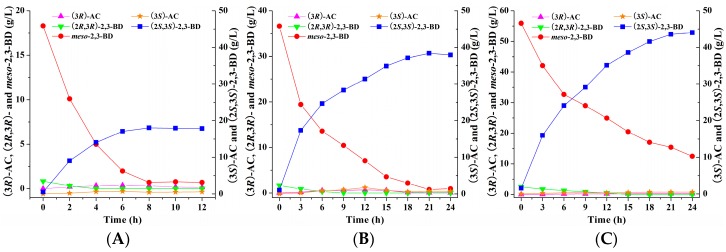
Time course of batch bioconversion of (2*S*,3*S*)-2,3-BD production from *meso*-2,3-BD with the initial substrate concentration of 20 (**A**); 40 (**B**); and 60 (**C**) g/L by synchronous catalysis in 5-L bioreactor.

Under optimal conditions, batch bioconversion by synchronous catalysis together with two biocatalysts was conducted in 5-L bioreactor with 2 L reaction mixture containing 20, 40, and 60 g/L 2,3-butanediol as substrate respectively. The results were shown in Figure 7. 44.03 g/L of (2*S*,3*S*)-2,3-butanediol was obtained from 60 g/L substrate at 24 h in the synchronous catalysis reaction. However, the residual *meso*-2,3-butanediol of 12.51 g/L was retained in the reaction, resulting in low (2*S*,3*S*)-2,3-butanediol stereoisomeric purity of 77.87%. On contrary, 18.04 and 38.41 g/L of (2*S*,3*S*)-2,3-butanediol with high stereoisomeric purity of 96.26% and 98.03% could be achieved from 20 and 40 g/L substrate at 8 h and 21 h respectively by synchronous catalysis. Considering the product yield and stereoisomeric purity, the initial substrate concentration of 40 g/L favored (2*S*,3*S*)-2,3-butanediol production in current reaction system. During the whole synchronous catalysis process, a small amount of (2*R*,3*R*)-2,3-butanediol in the 2,3-butanediol substrate could rapidly be consumed and no (2*R*,3*R*)-2,3-butanediol was detected at the end of synchronous catalysis. As reported in previous studies, (2*R*,3*R*)-2,3-butanediol was readily converted into (3*R*)-acetoin by the biocatalyst of *E. coli* (pET-*rrbdh*-*nox*-*vgb*) [22]. However, the (3*R*)-acetoin concentration was retained at low level in the whole synchronous catalysis reaction, implying that (3*R*)-acetoin from (2*R*,3*R*)-2,3-butanediol in the 2,3-butanediol substrate might be further converted into *meso*-2,3-butanediol by the biocatalyst of *E. coli* (pET-*ssbdh*-*fdh*). Our previous study showed that (2*S*,3*S*)-2,3-BDH from *Serratia* sp. T241 also catalyzed (3*R*)-acetoin into *meso*-2,3-butanediol [13], which could be used as substrate and converted into the product (2*S*,3*S*)-2,3-butanediol via (3*S*)-acetoin as an intermediate by induced *E. coli* (pET-*ssbdh*-*fdh*) cells during the synchronous catalysis process.

## 3. Materials and Methods

### 3.1. Enzymes and Chemicals

The standard samples of (3*S*/3*R*)-acetoin, (2*S*,3*S*)-2,3-butanediol (97.0%), (2*R*,3*R*)-2,3-butanediol (97.0%), and *meso*-2,3-butanediol (99.0%) were obtained from Sigma-Aldrich (Shanghai, China). The substrate 2,3-butanediol (93.73% *meso*-2,3-butanediol, 2.23% (2*S*,3*S*)-2,3-butanediol, and 4.04% (2*R*,3*R*)-2,3-butanediol) was purchased from Sinopharm (Beijing, China). Kanamycin was obtained from Amresco (Solon, OH, USA). Isopropyl-β-D-1-thiogalactopyranoside (IPTG) was purchased from Merck (Shainghai, China). PCR primers were synthesized by Sangon (Shanghai, China). *Pfu* DNA polymerase and restriction endonucleases were purchased from TaKaRa Biotech (Dalian, China). DNA, protein marker, and competent cells were obtained from Tiangen Biotech (Shanghai, China). All other chemicals were commercially available reagents of analytical grade.

### 3.2. Construction of the Recombinant Strains as Biocatalysts

The bacterial strains, plasmids, and primers used in this study are listed in Table 4. The genomic DNA of *P. polymyxa* ATCC12321, *L. brevis* 20054, *Serratia* sp. T241 and *C. boidinii* NCYC 1513 were extracted the OMEGA Bacterial Genomic DNA Kit (OMEGA, Shanghai, China). The *vgb* gene (GenBank Accession number AAA75506) source was from the pBR322-*vgb* plasmid previously constructed in our lab. For construction of the recombinant pET-*rrbdh* plasmid, the *rrbdh* gene (GenBank Accession number ADV15558) encoding (2*R*,3*R*)-2,3-BDH from *P. polymyxa* ATCC12321 were amplified by PCR using the primers P1/P2 with *Bam*HI and *Hin*dIII sites respectively. The obtained PCR products were inserted into the expression plasmid pET28a at the *Bam*HI and *Hin*dIII sites. ClonExpress MultiS One Step Cloning Kit (Vazyme, Nanjing, China) was used to develop the recombinant pET-*rrbdh*-*nox*, pET-*rrbdh*-*nox*-*vgb* and pET-*ssbdh*-*fdh* plasmids according to the protocol. In brief, a series of primers (P3–P6) were designed with adjacent oligos overlapped by 15–20 bp at each end of the assembly. The PCR-amplification products of the *rrbdh* and *nox* (GenBank Accession number AAN04047) genes using the primers P3/P4 and P5/P6 were assembled into the linearized pET28a vector at *Bam*HI and *Hin*dIII sites, generating the recombinant pET-*rrbdh*-*nox* plasmid. Similarly, the *rrbdh*, *nox*, *vgb*, *ssbdh* (GenBank Accession number AEF51363) and *fdh* (GenBank Accession number O13437) genes were amplified using the primers (P7–P16), respectively. The PCR-amplification products of the *rrbdh*, *nox* and *vgb* genes or the *ssbdh* and *fdh* genes were assembled into the linearized pET28a vector, resulting in the recombinant pET-*rrbdh*-*nox*-*vgb* and pET-*ssbdh*-*fdh* plasmids. Then, four recombinant plasmids of pET-*rrbdh*, pET-*rrbdh*-*nox*, pET-*rrbdh*-*nox*-*vgb*, and pET-*ssbdh*-*fdh* were transformed into competent cells of *E. coli* BL21 (DE3). The obtained recombinant strains were designated as *E. coli* (pET-*rrbdh*), *E. coli* (pET-*rrbdh*-*nox*), *E. coli* (pET-*rrbdh*-*nox*-*vgb*), and *E. coli* (pET-*ssbdh*-*fdh*) and kept at −80 °C for further experiments.

### 3.3. Biocatalyst Preparation

The recombinant strains harboring the plasmids pET-*rrbdh*, pET-*rrbdh*-*nox*, pET-*rrbdh*-*nox*-*vgb*, and pET-*ssbdh*-*fdh* were cultured in LB medium containing 50 μg/mL kanamycin at 37 °C until the OD_600_ reach up to 0.6, and then 0.5 mM IPTG was added into the culture for induction expression. After 8 h induction at 30 °C, the cells were harvested by centrifugation at 8000× *g* for 10 min at 4 °C and then washed twice with 0.85% NaCl. The cell pellets were resuspended in 50 mM potassium phosphate buffer for bioconversion experiments.

### 3.4. (3S)-Acetoin Production from meso-2,3-Butanediol by Whole-Cell Biocatalysis

The initial bioconversion experiments for (3*S*)-acetoin production from *meso*-2,3-butanediol by *E. coli* (pET-*rrbdh*), *E. coli* (pET-*rrbdh*-*nox*), and *E. coli* (pET-*rrbdh*-*nox*-*vgb*) were performed in 100 mL flasks containing 10 mL reaction mixtures (20 g/L 2,3-butanediol, 40 g WCW/L of induced cells, and pH 7.0) at 30 °C for 12 h, and *E. coli* (pET28a) was used as the control. For the optimization of bioconversion conditions, five factors including pH (6.0–10.0), temperature (25–42 °C), WCW (10–60 g/L), substrate concentration (20–160 g/L), and metal ions (Mn^2+^, Ca^2+^, Fe^2+^, Fe^3+^, and Mg^2+^ at 2.5 and 5.0 mM) were investigated using single factor experiments by *E. coli* (pET-*rrbdh*-*nox*-*vgb*). The substrate and product in the biocatalytic reactions were determined using gas chromatography. All the bioconversion experiments were performed in triplicate, and standard deviations of the biological replicates were represented by error bars.

### 3.5. (2S,3S)-2,3-Butanediol Production from meso-2,3-Butanediol by Synchronous Catalysis

Synchronous catalysis together with two biocatalysts of induced *E. coli* (pET-*rrbdh*-*nox*-*vgb*) cells and induced *E. coli* (pET-*ssbdh*-*fdh*) cells was used to evaluate the feasibility of (2*S*,3*S*)-2,3-butanediol production from *meso*-2,3-butanediol via (3*S*)-acetoin as an intermediate at 30 °C for 6 h. The initial reaction mixture contained phosphate buffer (pH 7.0), 20 g/L 2,3-butanediol, 20 g WCW/L of induced *E. coli* (pET-*rrbdh*-*nox*-*vgb*) cells, 20 g WCW/L of induced *E. coli* (pET-*ssbdh*-*fdh*) cells and 20 g/L ammonium formate as the driving force for NADH regeneration. The reaction was performed at 30 °C for 6 h. For the optimization of bioconversion conditions, four factors including pH (6.5–8.0), temperature (25–42 °C), the WCW ratio of *E. coli* (pET-*rrbdh*-*nox*-*vgb*) cells to *E. coli* (pET-*ssbdh*-*fdh*) cells (40 g/L:10 g/L to 40 g/L:40 g/L) and metal ions (Mn^2+^, Ca^2+^, Fe^2+^, Fe^3+^, and Mg^2+^ at 2.5 and 5.0 mM) were investigated using single factor experiments. All the bioconversion experiments were performed in triplicate, and standard deviations of the biological replicates were represented by error bars.

### 3.6. Enzyme Assays

The induced cells after harvest by centrifugation were resuspended in 50 mM potassium phosphate buffer (pH 7.4) and disrupted by sonication in an ice bath for 10 min. The disrupted cells were centrifuged at 10,000× *g* for 10 min at 4 °C to remove the cell debris, and the obtained supernatant was used as the crude enzyme extract for enzyme activity assays. The enzyme activities of (2*R*,3*R*)-2,3-BDH and FDH were assayed by measuring the changes in absorbance at 340 nm corresponding to the reduction of NAD^+^ using a UV/visible spectrophotometer (UV-1800, Mapada, Shanghai, China) [22,33]. The reaction mixtures containing 50 mM potassium phosphate buffer (pH 8.5), 0.2 mM NAD^+^ and 50 mM 2,3-butanediol ((2*S*,3*S*)-2,3-butanediol or (2*R*,3*R*)-2,3-butanediol or *meso*-2,3-butanediol) were used to determine (2*R*,3*R*)-2,3-BDH activity. The assay of FDH activity was carried out in 50 mM potassium phosphate buffer (pH 7.0) with 0.2 mM NAD^+^ and 100 mM formate as substrate. The enzyme activities of (2*S*,3*S*)-2,3-BDH and NOX were assayed by measuring the changes in absorbance at 340 nm corresponding to the oxidation of NADH using a UV/visible spectrophotometer (UV-1800, Mapada, Shanghai, China) [13,34]. The reaction mixtures for (2*S*,3*S*)-2,3-BDH activity assay contained 50 mM potassium phosphate buffer (pH 7.0), 0.2 mM NADH and 50 mM (3*R*/3*S*)-acetoin as substrate. The NOX activity assay was carried out in 50 mM potassium phosphate buffer with 0.2 mM NADH as the substrate. One unit of (2*R*,3*R*)-2,3-BDH and FDH activity was defined as the amount of enzyme required to reduce 1 μmol of NAD^+^ in one minute. One unit of (2*S*, 3*S*)-2,3-BDH and NOX activity was defined as the amount of enzyme required to oxidize 1 μmol of NADH in one minute. All enzyme activities were determined in triplicate, and standard deviations of the biological replicates were represented by error bars. The protein concentration was determined by the Bradford method using bovine serum albumin as the standard.

### 3.7. Product Analysis

The samples in the bioconversion reactions were centrifuged at 10,000× *g* for 5 min. The concentration of acetoin and 2,3-butanediol in the supernatant was analyzed by a GC system (Agilent 7820A, Santa Clara, CA, USA), but before GC analysis, the supernatant was extracted by ethyl acetate with the addition of *n*-butanol as the internal standard. The GC system consisted of a FID detector and a chiral column (Supelco β-DE 120, 30-m length, and 0.25-mm inner diameter; Sigma-Aldrich). The operation conditions were as follows: N_2_ was used as the carrier gas at a flow rate of 1.2 mL/min; the injector temperature and the detector temperature were 215 °C and 245 °C, respectively; and the column temperature was maintained at 50 °C for 1.5 min, then raised to 180 °C at a rate of 15 °C/min. The concentration of acetoin and 2,3-butanediol in the supernatant was determined using standard curves [5,35].

## 4. Conclusions

In summary, a novel *E. coli* (pET-*rrbdh*-*nox*-*vgb*) biocatalyst was developed for efficiently catalyzing the stereospecific oxidation of *meso*-2,3-butanediol to (3*S*)-acetoin. Maximum (3*S*)-acetoin concentration of 72.38 g/L with the productivity of 3.02 g/L/h and stereoisomeric purity of 94.65% was obtained by induced *E. coli* (pET-*rrbdh*-*nox*-*vgb*) cells under optimal conditions. Subsequently, the strategy of synchronous catalysis was established for efficient (2*S*,3*S*)-2,3-butanediol production from *meso*-2,3-butanediol via (3*S*)-acetoin as an intermediate through using two biocatalysts of *E. coli* (pET-*rrbdh*-*nox*-*vgb*) and *E. coli* (pET-*ssbdh*-*fdh*). Under optimal conditions, 38.41 g/L (2*S*,3*S*)-2,3-butanediol with stereoisomeric purity of 98.03% was achieved from 40 g/L 2,3-butanediol by synchronous catalysis. These results suggested that biocatalysis was a powerful and effective strategy for the production of enantiomerically pure building blocks that cannot be achieved through chemical and fermentation processes.

## Figures and Tables

**Table 1:** Activity assays of (2*R*,3*R*)-2,3-BDH and NADH oxidase in the recombinant *E. coli* strains.

*E. coli* BL21(DE3)	Crude Enzyme Activities of (2*R*,3*R*)-2,3-BDH and NADH Oxidase (U/mg)			
	(2*S*,3*S*)-2,3-BD	(2*R*,3*R*)-2,3-BD	*meso*-2,3-BD	NADH
pET28a	0	0.01 ± 0.01	0.04 ± 0.01	ND
pET-*rrbdh*	0	1.11 ± 0.04	0.57 ± 0.03	ND
pET-*rrbdh*-*nox*	0	0.97 ± 0.05	0.53 ± 0.03	6.83 ± 0.48
pET-*rrbdh*-*nox*-*vgb*	0	0.95 ± 0.02	0.51 ± 0.04	5.93 ± 0.35

ND: not detected.

**Table 2:** Activity assays of (2*S*,3*S*)-2,3-BDH and FDH in the recombinant *E. coli* strains.

*E. coli* BL21(DE3)	Crude Enzyme Activities of (2*S*,3*S*)-2,3-BDH and FDH (U/mg)	
	(3R/3S)-AC	Formate
pET28a	0.05 ± 0.01	ND
pET-*ssbdh*	53.20 ± 0.49	ND
pET-*fdh*	0.03 ± 0.01	0.18 ± 0.01
pET-*ssbdh*-*fdh*	38.90 ± 0.21	0.06 ± 0.02

ND: not detected.

**Table 3:** The feasibility of (3*S*)-AC production from *meso*-2,3-BD by the recombinant strains.

*E. coli* BL21(DE3)	(3*R*)-AC (g/L)	(3*S*)-AC (g/L)	(2*S*,3*S*)-2,3-BD (g/L)	(2*R*,3*R*)-2,3-BD (g/L)	*meso*-2,3-BD (g/L)
pET28a	0.06 ± 0.01	0.04 ± 0.01	0.52 ± 0.03	0.86 ± 0.02	18.75 ± 0.19
pET-*rrbdh*	0.71 ± 0.03	7.86 ± 0.05	0.53 ± 0.02	0.27 ± 0.01	10.46 ± 0.14
pET-*rrbdh*-*nox*	0.81 ± 0.05	13.88 ± 0.12	0.55 ± 0.03	0.12 ± 0.01	4.52 ± 0.10
pET-*rrbdh*-*nox*-*vgb*	0.83 ± 0.02	16.79 ± 0.15	0.55 ± 0.01	0.09 ± 0.03	1.96 ± 0.05

**Table 4:** Bacterial strains, plasmids, and primers used in this study.

Strains, Plasmids, or Primers	Genotype, Properties, or Sequences	Source
Strains		
*P. polymyxa* ATCC12321	Wild type	Laboratory stock
*L. brevis* DSM 20054	Wild type	Laboratory stock
*Serratia* sp. T241	Wild type	Laboratory stock
*C. boidinii* NCYC 1513	Wild type	Laboratory stock
*E. coli* DH5α	F^−^, φ80d/*lac*ZΔM15, Δ(*lacZYA-argF*)U169, *deoR*, *re*cA1, *endA*1, *hsdR*17(*r*k^−^*m*k^+^), *phoA*, *supE*44, λ^−^, *thi*-1, *gyrA*96, *relA*1	Tiangen Biotech
*E. coli* BL21(DE3)	F-, ompT, hsdSB(rB-mB-), gal(λ c I 857, ind1, Sam7, nin5, lacUV-T7 gene1), dcm(DE3)	Tiangen Biotech
Plasmids		
pET28a	Km^r^; expression vector	Laboratory stock
pBR322-*vgb*	AMP^r^; pBR322 vector containing the *vgb* gene	Laboratory stock
pET-*fdh*	Km^r^; *fdh* in pET28a	Laboratory stock
pET-*ssbdh*	Km^r^; *ssbdh* in pET28a	Laboratory stock
pET-*rrbdh*	Km^r^; *rrbdh* in pET28a	This study
pET-*rrbdh*-*nox*	Km^r^; *rrbdh* and *nox* in pET28a	This study
pET-*rrbdh*-*nox*-*vgb*	Km^r^; *rrbdh*, *nox*, and *vgb* in pET28a	This study
pET-*ssbdh*-*fdh*	Km^r^; *rrbdh* and *fdh* in pET28a	This study
Primers		
P1	CGCGGATCCATGCAAGCATTGAGATGGC	*Bam*HI
P2	CGCAAGCTTTTAGGCTTTCGGAGATACCA	*Hin*dIII
P3	CAGCAAATGGGTCGCGGATCCATGCAAGCATTGAGATGGC	*Bam*HI
P4	TTTCCTTTCCTTATTGTGATGACTTAGGCTTTCGGAGATAC	RBS sequence
P5	GTCATCACAATAAGGAAAGGAAAATGAAAGTCACAGTTG	RBS sequence
P6	CTCGAGTGCGGCCGCAAGCTTTTAAGCGTTAACTGAT	*Hin*dIII
P7	CAGCAAATGGGTCGCGGATCCATGCAAGCATTGAGATGGC	*Bam*HI
P8	TTTCCTTTCCTTATTGTGATGACTTAGGCTTTCGGAGATAC	RBS sequence
P9	GTCATCACAATAAGGAAAGGAAAATGAAAGTCACAGTTG	RBS sequence
P10	TTTCCTTTCCTTATTGTGATGACTTAAGCGTTAACTGAT	RBS sequence
P11	GTCATCACAATAAGGAAAGGAAAATGTTAGACCAGCAAAC	RBS sequence
P12	CTCGAGTGCGGCCGCAAGCTTTTATTCAACCGCTTGAGCGT	*Hin*dIII
P13	CAGCAAATGGGTCGCGGATCCATGTCGACAGGTTTGAAC	*Bam*HI
P14	TTTCCTTTCCTTATTGTGATGACTTAGCGATAAACCAGCCC	RBS sequence
P15	GTCATCACAATAAGGAAAGGAAAATGAAAATTGTCCTGGT	RBS sequence
P16	CTCGAGTGCGGCCGCAAGCTTTTACTTTTTATCGTGTTTG	*Hin*dIII
